# Trade-offs between parameter constraints and model realism: a case study

**DOI:** 10.1038/s41598-019-46963-6

**Published:** 2019-07-24

**Authors:** Florian U. Jehn, Alejandro Chamorro, Tobias Houska, Lutz Breuer

**Affiliations:** 1 Institute for Landscape Ecology and Resources Management (ILR), Research Centre for BioSystems, Land Use and Nutrition (iFZ), Justus Liebig University Giessen, Heinrich-Buff-Ring 26, 35390 Giessen, Germany; 2 Centre for International Development and Environmental Research (ZEU), Justus Liebig University Giessen, Senckenbergstrasse 3, 35392 Giessen, Germany

**Keywords:** Hydrology, Environmental sciences

## Abstract

Tightly constraint parameter ranges are seen as an important goal in constructing hydrological models, a difficult task in complex models. However, many studies show that complex models are often good at capturing the behaviour of a river. Therefore, this study explores the trade-offs between tightly constrained parameters and the ability to predict hydrological signatures, that capture the behaviour of a river. To accomplish this we built five models of differing complexity, ranging from a simple lumped model to a semi-lumped model with eight spatial subdivisions. All models are built within the same modelling framework, use the same data, and are calibrated with the same algorithm. We also consider two different methods for the potential evapotranspiration. We found that that there is a clear trade-off along the axis of complexity. While the more simple models can constrain their parameters quite well, they fail to get the hydrological signatures right. It is the other way around for the more complex models. The method of evapotranspiration only influences the parameters directly related to it. This study highlights that it is important to focus not only on parametric uncertainty. Tightly constrained parameters can be misguiding as they give credibility to oversimplified model structures.

## Introduction

How complex should a hydrological model be? This question is still unsolved in hydrology. Recent advancements in experimental hydrology provide more data and lead to a better understanding of hydrology. This additional data and knowledge could be used to build more complex hydrological models. However, it is questioned if this will lead to better models. A higher complexity means more parameters and more parameters lead to a more difficult calibration^1^ and are sometimes seen as the main source of uncertainty^2^. Besides the parametric uncertainty, different kinds of uncertainty sources exist. Namely, model structure, evaluation, and forcing data. All of which are treated differently depending on the choice of the objective function and the calibration scheme applied. As uncertainty causes so many problems, like potentially undermining the trustworthiness or decreasing the forecasting ability of models^3^, it is sometimes referred to as the biggest problem in hydrology^4^.

However, hydrological models need to incorporate at least those hydrological features of a landscape that are needed to reflect dominant hydrological processes^5–8^. To address this, it has become common practice to compare a range of models of different complexity. Complexity of the model structure in this context refers to the amount of processes in a model structure and its spatial subdivision (lumped vs semi-distributed vs distributed). Thus a model structure is more complex when it includes more processes and/or has a finer spatial resolution^9–12^. Those studies examine the influence of model structure and spatial layout and come to contrasting results. Some find that there is no difference between lumped and semi distributed models^10^, the lumped ones perform better^13^ or the (semi) distributed perform better^7^.

Those problems of model structural uncertainty get aggravated as models are often compared without an underlying framework. This hinders comparisons of model structures and implemented processes^14^. This was also noted by other authors^13^, who state that many comparisons of lumped and semi-distributed models are hindered by different selections of included processes. This can be avoided by using a fixed modelling framework, which standardizes all steps of model structure development. In such a framework, all models are treated the same way, so that the differences in performance between models are only caused by the model structure itself.

Looking at the statements above it becomes clear that hydrological modellers are in a dilemma. Their models should avoid over-parametrization, but their models should also include all relevant hydrological processes. This implies a trade-off between the realism of the model and its ability to constrain its parameters. To explore this dilemma, this study will look at five different model structures, ranging from a simple lumped model to a semi-lumped model that takes vegetation and topography into account. All of those five models are run with two different methods to calculate the potential evapotranspiration (PET). PET has been identified as one very important process in models of this complexity concerning the simulation of discharge^9^, and it is still not clear if simple temperature-based calculations can better help constraining the parameters of a model or not^15–17^. To ensure comparability^13^, all models are built with the Catchment Modelling Framework (CMF)^18,19^. CMF is one of the few existing modelling frameworks that allows the isolation of the effects of the model structures and processes like the PET. The ROPE algorithm^20^ is used to calibrate the models, as it is capable of generating parameter sets with a small range of potential parameter values^20^. Using those tools, the aim of this study is to explore the trade-offs between the ability of a model structure to constrain its parameters, and the realism of the model structure. Realism is expressed as the performance of a model to simulate a variety of hydrologic signatures^21,22^ for which the model has not been calibrated.

## Materials and Methods

### Study area

**Figure 1:**
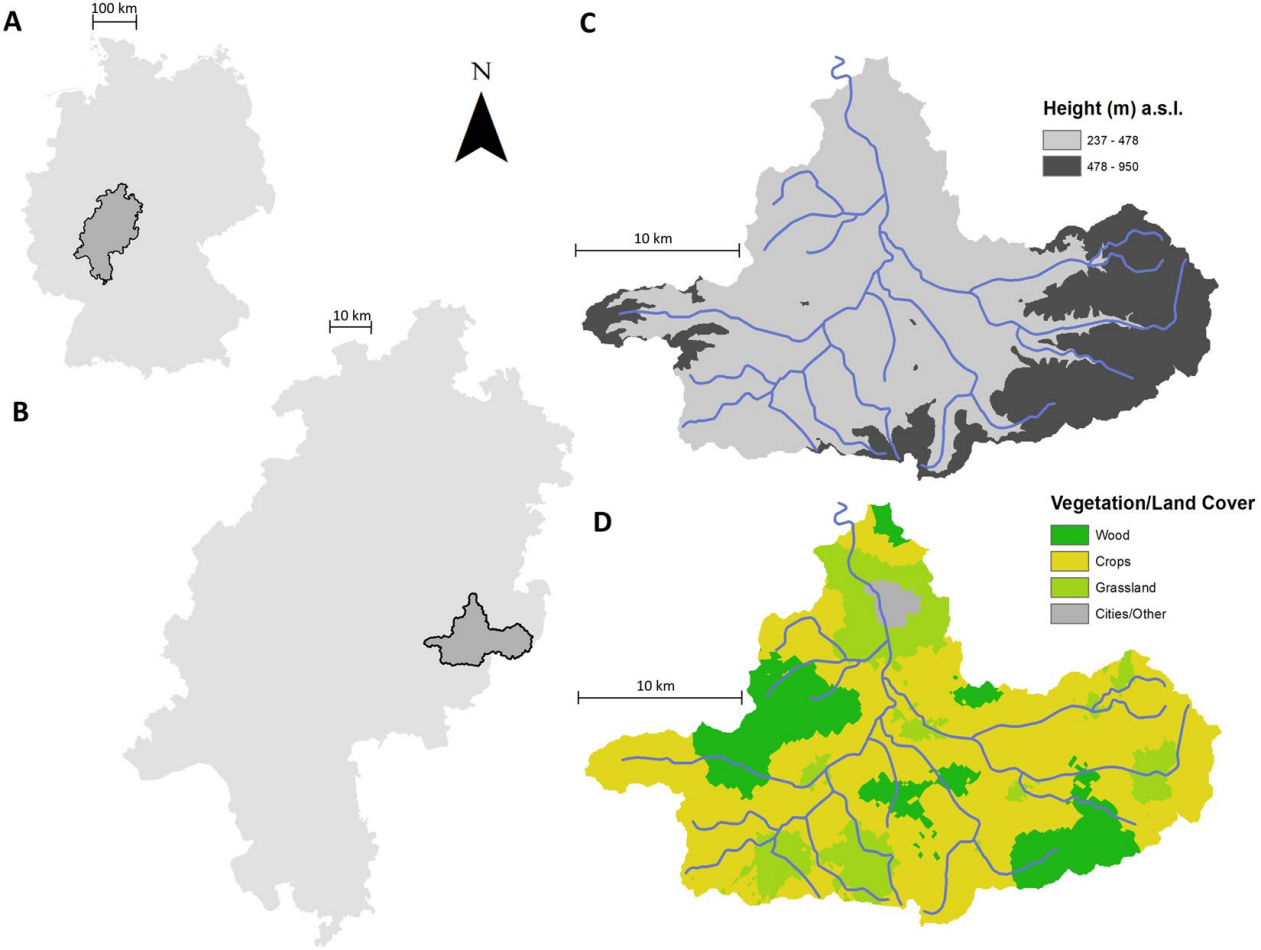
Location of Hesse in Germany (**A**), Location of the Fulda catchment in Hesse (**B**) (gauging station Kämmerzell) and separation of the catchment by height (**C**) and vegetation/land cover (**D**).

The study area is the upper part of the Fulda catchment (Catchment area 562 km², gauging station Kämmerzell). The catchment has Mid-European temperate climatic conditions. To the east and west, the river receives water from two ridges: the Wasserkuppe and the Vogelsberg. Elevation ranges from 237 m a.s.l. to 950 m a.s.l. Land use is dominated by agriculture (~50%) and forests (~40%) (Fig. 1). For more details see Jehn *et al*.^23^.

Meteorological data for model forcing and discharge data for model calibration and validation are obtained from the Hessisches Landesamt für Naturschutz, Umwelt und Geologie (HLNUG, https://www.hlnug.de/messwerte.html) for the period 1979–1989. The discharge is measured at the Kämmerzell gauging station. Windspeed, relative humidity, sunshine duration, and temperature are taken from nine weather stations located in close vicinity to the catchment (Eschwege, Wasserkuppe, Grebenhain, Melsungen, Wartenberg, Neukirchen, Kassel, Bad Hersfeld and Fulda). Both the model time step and the temporal resolution of the input data are daily. This is in line with recommended temporal resolution based on results obtained for mesoscale model applications^24^.

### Model framework

All models were constructed using the open source, modular Catchment Modelling Framework (CMF)^19^. Additional information can be found at the framework’s website^18^. To avoid numerical problems^25^, we selected the CVode Integrator^26^ as the numerical solver. The CMF version used for this study was 1.1.1.

**Figure 2:**
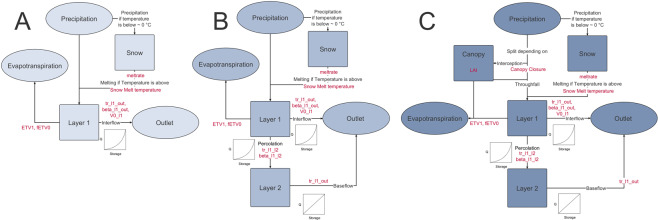
Model structure for Lumped 1 (**A**), Lumped 2 (**B**) and Lumped 3 and Semi-Lumped 3 (**C**). Calibration parameters shown in red.

The base model structure consists of a one storage set up with a simple snow storage and actual evapotranspiration (Fig. 2). The storage receives precipitation when it is warmer than 0 °C. Otherwise, the precipitation is stored as snow. Water in the storage gets either evapotranspirated or is transferred to the outlet. Following the findings of Singh^27^, all connections in the model (Fig. 2) are described as kinematic waves (Eq. 1):

1 $$Q={Q}_{0}{(\frac{V-{V}_{residual}}{{V}_{0}})}^{\beta }$$

where *Q* is the amount of water transferred from one storage to the other, *V*_*residual*_ [m³] is the volume of water remaining in the storage at each time step, *V*_0_ [m³] is the reference volume (calibrated) to scale the exponent, *V* is the current volume of water in the storage [m³] at each time step, and *β* is a parameter to shape the response curve [−]. *Q*_0_ is the flux in [m³ d^−1^], when $\frac{V-{V}_{residual}}{{V}_{0}}=1$.

The code for all models is freely available on GitHub and is stored in a citable repository^28^. In the following it will be explained how this base structure is built upon to create the more complex models.

#### Model structures

A total of five model structures were constructed, three lumped and two semi-lumped models. Semi-lumped is used here in line with in Andréassian *et al*.^13^, meaning a lumped model with a spatial subdivision, but with the same parameters for each spatial subdivision. The models differ in three complexities (1–3). While the most simple lumped model Lumped 1 consists of only one storage *Layer 1* (Fig. 2), evapotranspiration and a snow storage (7 parameters), the moderate complex lumped model Lumped 2 uses an second storage *Layer 2* (10 parameters). In addition to this, the most complex lumped model Lumped 3 features a simulation of the canopy storage *Canopy* (12 parameters). A detailed description of the parameters is given in Table 1. The number of parameters is similar to other studies that compared models of differing complexity^9^.

For the two semi-lumped models we used the model structure of the most complex lumped model Lumped 3. The spatial subdivision for the first semi-lumped model Semi-Lumped 3-Vegetation is based on vegetation (forest, arable land, grassland and settlements/other) (Fig. 1). For the second semi-lumped model Semi-Lumped 3-Vegetation/Height an additional split between high (above 478 m a.s.l.; 25% of the catchment) and low (equal or below 478 m a.s.l.; 75% of the catchment) elevation was considered, resulting in eight spatial subdivisions. For those spatial subdivisions, the point measurements for the forcing data were interpolated, using external drift kriging with the height as external drift. For the lumped models, the interpolated data was arithmetically averaged for the whole catchment. In case of the semi-lumped models, the interpolated data were split into the separate spatial subdivisions, and the averages were calculated separately. This was necessary to bring the data in an appropriate format for the semi-lumped models.

#### Potential evapotranspiration

In addition, every model exists in two versions, depending on the methodology used for the calculation of the PET. For this, we considered the methods according to Hargreaves^29^ and Penman-Monteith^30^ (also referred to as Penman). A detailed description of the calculation of the PET methods can be found in the Supplementary Information.

**Table 1:** Parameter for all models with their intended meaning and ranges considered during calibration. Parameter related processes are shown in Fig. 1.

Name	Unit	Intended meaning	Model Structure	Min	Max
tr_l1_l2	day	Residence time from layer 1 to layer 2	B, C	1	400
tr_l1_out	day	Residence time from layer 1 to outlet	A, B, C	1	200
tr_l2_out	day	Residence time from layer 2 to outlet	B, C	1	650
V0_l1	mm	Field capacity of the soil	A, B, C	1	300
beta_l1_l2	—	Exponent the changes the shape of the flow curve	B, C	0.5	6
beta_l1_out	—	Exponent the changes the shape of the flow curve	A, B, C	0.3	8
ETV1	mm	Volume under which the evapotranspiration is lowered	A, B, C	1	300
fETV0	%	Factor by what the evapotranspiration is lowered	A, B, C	0	0.9
meltrate	mm °C^−1^ day^−1^	Melt rate of the snow	A, B, C	0	12
snow_melt_temp	°C	Temperature of snow melt	A, B, C	−3	3
LAI	—	Leaf area index	C	1	12
CanopyClosure	%	Canopy closure	C	0.1	0.9

### Calibration and validation

The models were calibrated using the ROPE algorithm^20^, as implemented in the SPOTPY package^31^. The algorithm itself was run 100,000 times. For further analysis the 1,000 best runs of the last set were used, as proposed Bardossy and Singh^20^. The performance of all models was evaluated using the Kling-Gupta Efficiency (KGE)^32^. The time series was split into a warm up period (1979), the calibration (1980–1984), and validation period (1985–1989).

All parameters (Table 1) were sampled from a uniform distribution. The ranges for V0 and ETV1 were in agreement with typical field capacity values for German soils^33^, while canopy parameters were taken from Breuer *et al*.^34^. All other parameters were subjectively set, as their conceptual nature does not allow to link them directly to physical processes. However, their ranges were in line with other studies that explored the Fulda catchment using models^23,35^ and field experimental approaches like tritium^36^.

### Model evaluation

The realism of all models was subsequently evaluated by how much it was possible to constrain their parameters and their ability to correctly simulate a selection of hydrological signatures, which they were not calibrated for (Table 2). This way of assessing the models realism allows to evaluate both, their ability to constrain parameters and the realism of their simulations.

**Table 2:** Hydrological signatures used in this study were taken from Westerberg and McMillan (2015). All signatures are calculated on daily data and for the whole time period.

	Signature	Name	Description	Unit
Flow distribution	Q_mean_	Mean flow	Mean flow for the analysis period	mm d^−1^
	Q_0.01_, Q_99_	Flow percentiles	Low- and high-flow exceedance percentiles from the flow duration curve (FDC)	mm d^−1^
Event frequency and duration	Q_HF_	High-flow event frequency	Average number of daily high-flow events per year with a threshold of 9 times the median daily flow^58^	yr^−1^
	Q_HD_	High-flow event duration	Average duration of daily flow events higher days than 9 times the median daily flow^58^	days
	Q_LF_	Low-flow event frequency	Average number of daily low-flow events per year with a threshold of 0.2 times the mean daily flow^59^	yr^−1^
	Q_LD_	Low-flow event duration	Average duration of daily flow events lower days than 0.2 times the mean daily flow^59^	days
Flow dynamics	BFI	Base-flow index	Contribution of base flow to total streamflow calculated from daily flows using the Flood Estimation Handbook method^60^	—
	S_FDC_	Slope of normalized FDC	Slope of the FDC between the 33 and 66% exceedance values of streamflow normalized by its mean^61^	—
	Q_CV_	Overall flow variability	Coefficient of variation in streamflow, i.e. standard deviation divided by mean flow^58,62^	—
	Q_LV_	Low-flow variability	Mean of annual minimum flow divided by the median flow^62^	—
	Q_HV_	High-flow variability	Mean of annual maximum flow divided by the median flow^62^	—
	Q_AC_	Flow autocorrelation	Autocorrelation for 1 day (24 h)^21,63,64^	—

The parameter distribution is evaluated by comparing the parameters before and after calibration. A range reduction factor is determined to indicate how much those differ in their range [in %]. We choose the constraint of the parameters as one criteria in this study, as unconstrained parameters are often stated as a core problem in hydrology^1,2^.

For the hydrological signatures, we selected a number of those signatures presented by Westerberg and McMillan^22^ (Table 2). Those signatures capture the behaviour of a river concerning its flow distribution (high, mean and low flows), the frequency and duration of high and low flow events and the dynamics of the flow. They are widely used for catchment classification, and model calibration^22^. The signatures were calculated for the whole time period on daily data. We choose hydrological signatures to assess the realism of the simulation, as in recent years hydrological signatures are used more and more often to detect weaknesses in hydrological models^21,37^.

## Results

### Model performance

**Figure 3:**
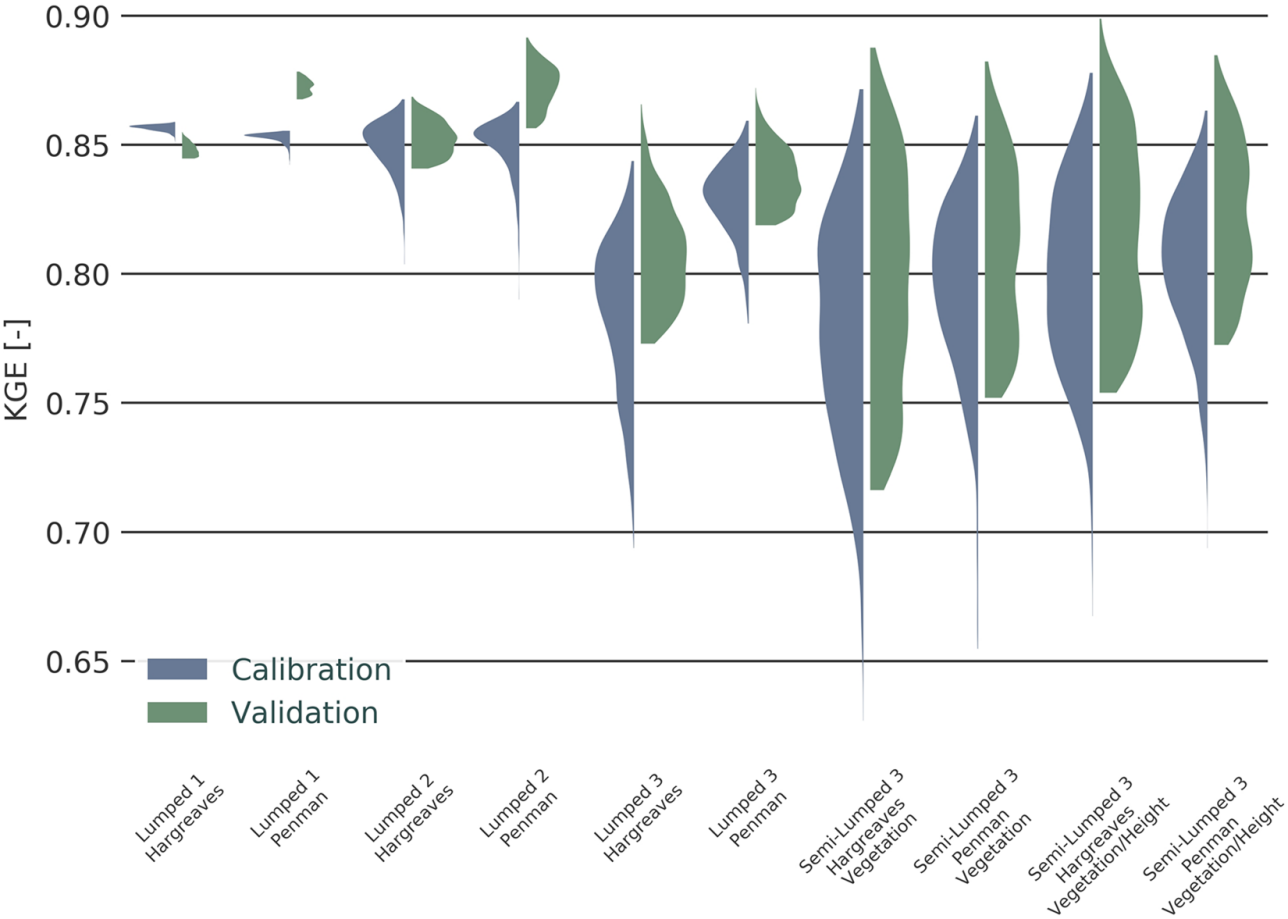
Model performance according to the Kling-Gupta-Efficiency (KGE) for all models, seperated by the calibration and validation period.

All models were able to produce runs that have KGEs above 0.8. In addition, all models performed better in the validation than in the calibration period (Fig. 3), with the exception of the model Lumped 3 Hargreaves. The semi-lumped models reach slightly higher maximal KGE values than the lumped models. However, the semi-lumped models in combination with the Hargreaves PET method also show the overall largest spread and the lowest KGEs values. This tendency of a comparatively large KGE spread is also found for the more complex Lumped 3 models. For the more simple models Lumped 1 and particular for Lumped 2 it is the other way around. Here the models with the Penman PET method have a marginally larger spread of the objective function.

### Parameter constraints

**Figure 4:**
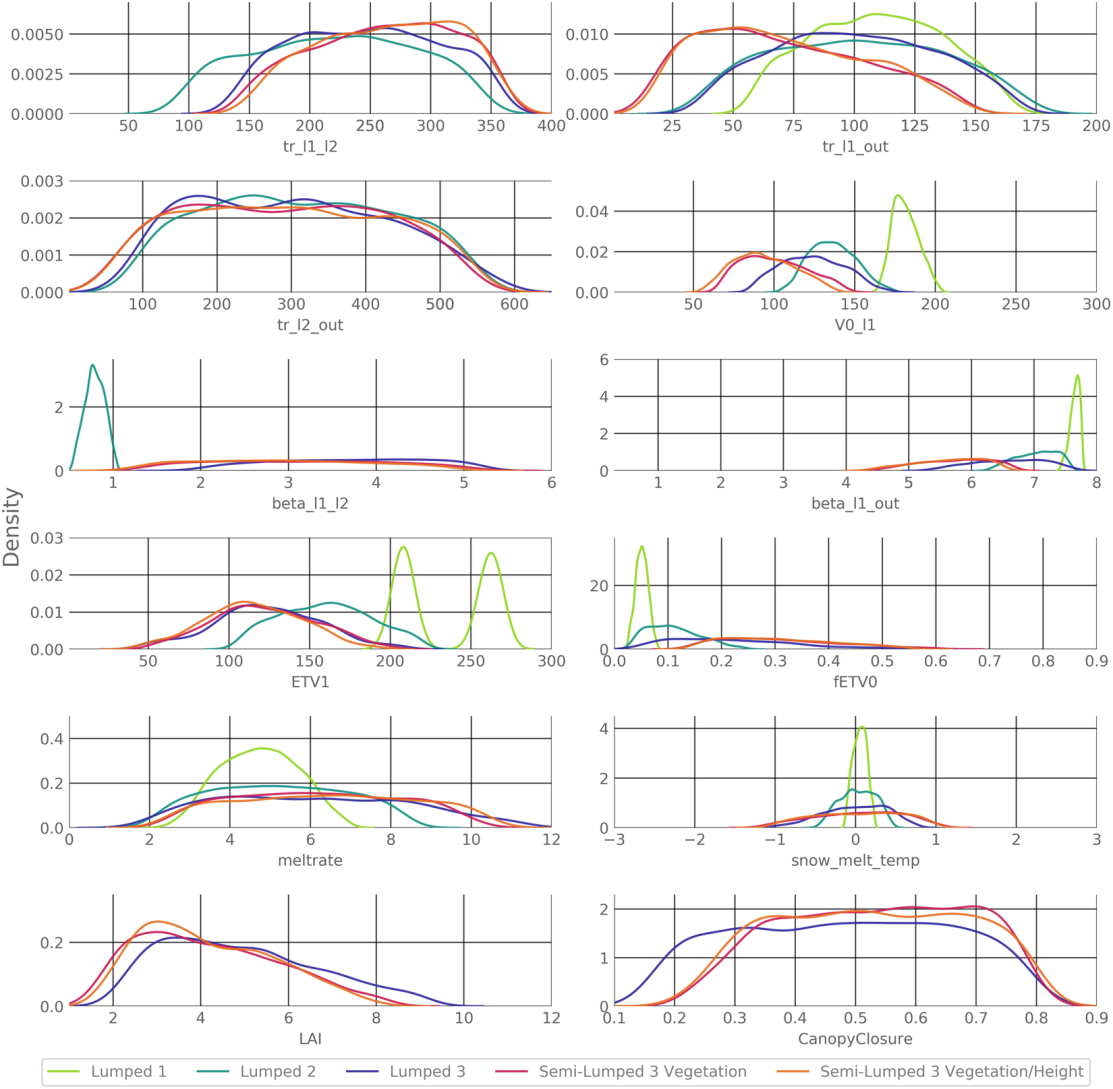
Posterior parameter distribution separated by model structures shown in different coloured lines. Different PET calculations for a model structure are pooled. X-axes scales equal the *a priori* distribution of the parameters before calibration. Lines are fitted with a Gaussian kernel density function.

**Figure 5:**
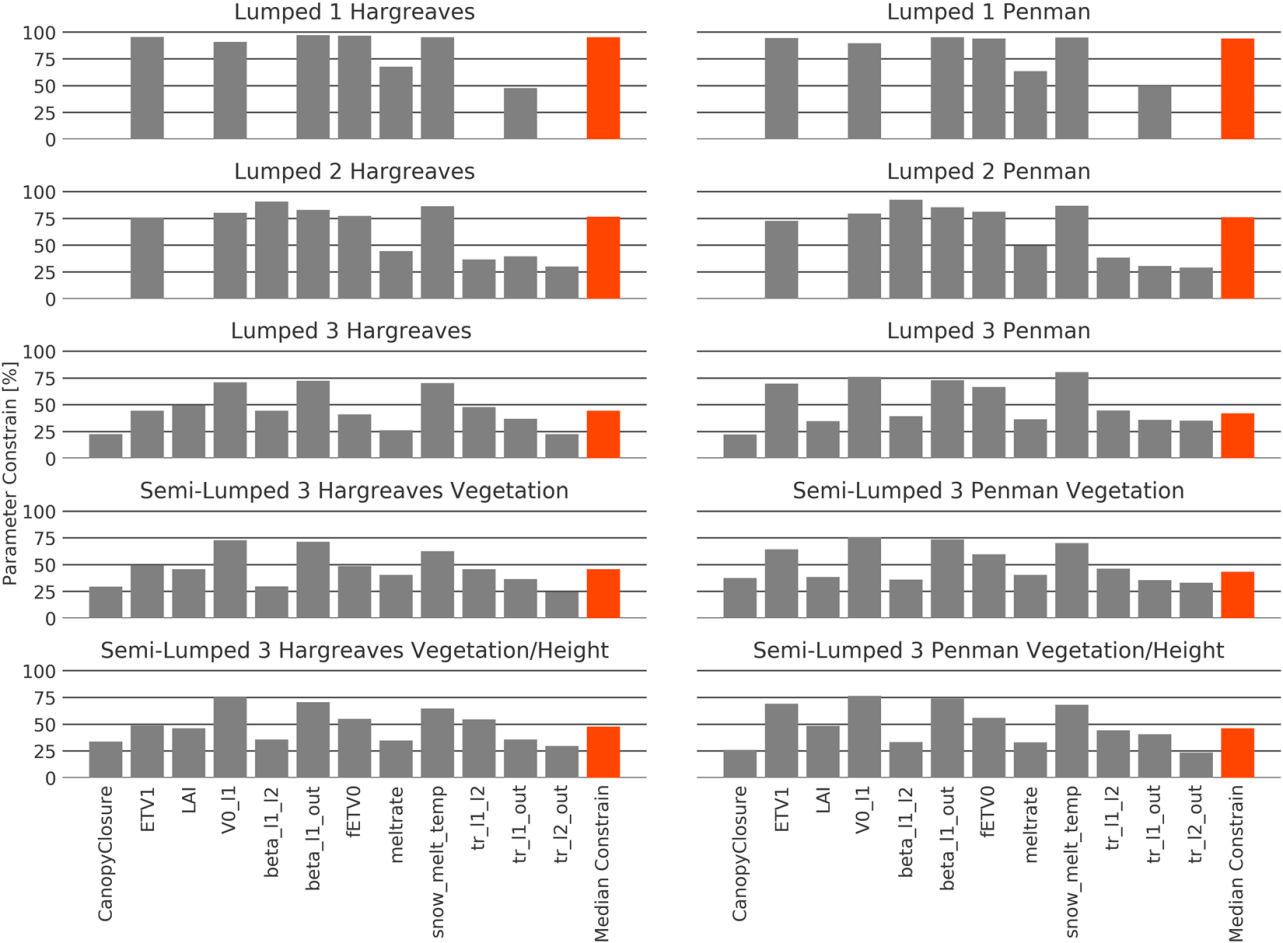
Parameter constrainability for all model structures separated by parameters. Red bar marks the median parameter constrainability for each model. Larger bars indicate larger constrained parameters. Parameter constrainability is defined as the difference between the range of the parameter before and after the calibration in percent.

When looking at the parameter distribution for all single model structures, the simpler models show a smaller range in the parameter distribution (Fig. 4). Lumped 1 is the model structure that is most able to constrain its parameters. This is true for both PET version, with a median parameter constraint of 95% (Fig. 5). All other model structures are less able to constrain their parameters (Figs 4 and 5). Especially the model structures Lumped 3 and Semi-Lumped 3 both have a median parameter constraint below 50% and contain parameters like *tr_l2_out* (Residence time from layer 2 to outlet), which can only be constrained by 25%.

The ability of the different model structures to constrain a parameter is also highly dependent on the parameter itself. We find three classes of parameters. Parameters like *V0_l1* (field capacity of the soil) or *snow_melt_temp* (temperature of the snow melt) have a very clear peak in the distribution after the calibration and are constrainable. Other parameters such as *tr_l2_out* (transition time from lower layer to outlet) or other residence time parameters are difficult to be constrained at all. A third class of parameters like *fETV0* (reduction of the PET under dry conditions) and *beta_l1_l2* (shapes the flow curve) show an ambiguous behaviour with better constrainability for the lumped model structures. Overall parameters, which can be constrained best by the models, are related to the evapotranspiration, the snow melt, and the water flux from the first layer to the outlet. Parameters related to the second layer and the canopy structure cannot be constrained well by the different model structures.

**Figure 6:**
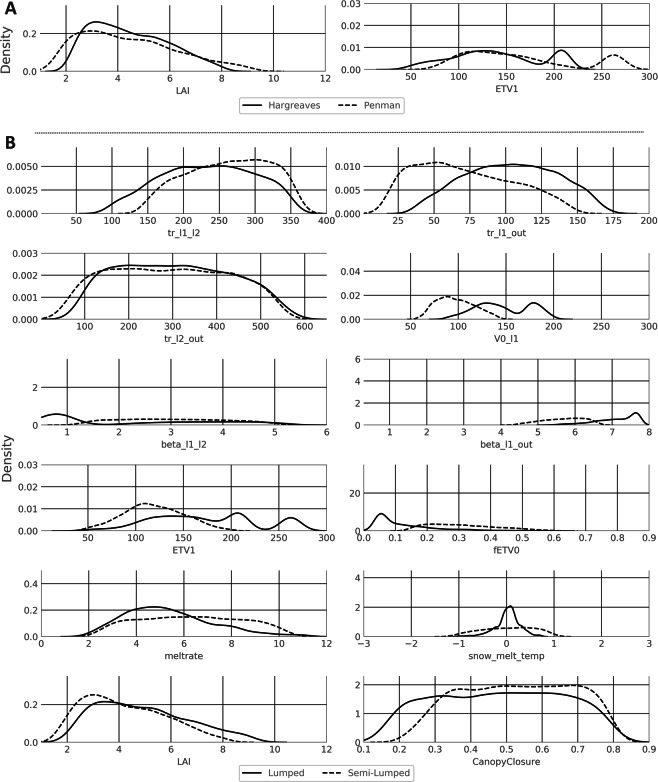
Posterior parameter distributions separated by PET method for the parameters influenced by PET method. Different model complexities are pooled (**A**). And distributions separated by spatial subdivision for the parameters influenced by spatial subdivision. Different model complexities are pooled (**B**). X-axes scales equal the *a priori* distribution of the parameters before calibration. Lines are fitted with a Gaussian kernel density function.

The distributions of the parameters are influenced more by the spatial subdivision than by the PET (Fig. 6A). When all model structures are pooled and only the difference between Hargreaves and Penman is considered (Fig. 6A), the only parameter where larger differences can be found is *ETV1* (Volume below which the PET is lowered by *fETV0*). For *ETV1* the models with Penman have a peak in the distribution of the parameter at around 270 [mm], while the Hargreaves models peak at 210 [mm]. The second parameter that is influenced by the PET is the *LAI* parameter. The peak in the distribution of *LAI* is slightly shifted to the left for the Penman models in comparison with the Hargreaves models.

The differences become clearer when all lumped and semi-lumped models are pooled (Fig. 6B). Here most parameters show at least some deviations. Parameters like *V0_l1* or *ETV1* even depicting very different distributions. The only parameter that experiences a shift in both comparisons (lumped vs semi-lumped and Hargreaves vs Penman) is *ETV1* and to some extent the *LAI*. While the shape of the distribution for the *LAI* has its peak at around 3.5 for both the PET method and the comparison between lumped and semi-lumped, *EVT1* shows a different shape of the distribution. The distinction is clearer in the comparison of the lumped and semi-lumped models. The unimodal distribution for the semi-lumped models is very different to the bimodal distribution of the lumped models.

### Model realism in regard to hydrological signatures

**Figure 7:**
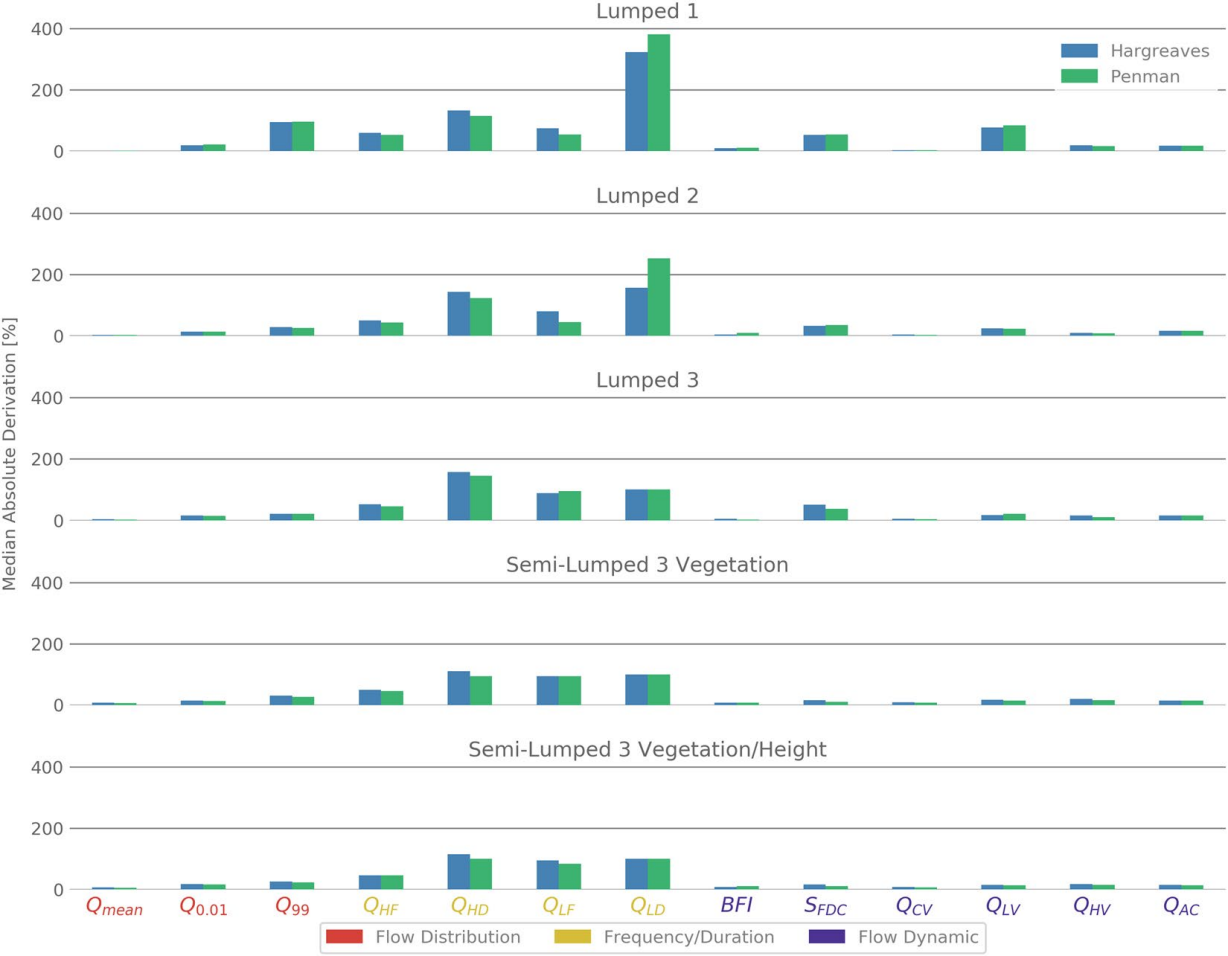
Median absolute deviations (%) of simulated versus observed hydrological signatures. Smaller values indicate smaller error in the simulation.

In the next step, we challenge the various model structures to simulate a large set of hydrological signatures, and relate their performance to the information on parameter distribution and KGEs. The simulated hydrological signatures (explanation of signatures in Table 2) shown in Fig. 7 depict different model performances compared to the previously described parameter distributions and KGEs. The simpler models, especially Lumped 1 and Lumped 2, are able to achieve consistently high KGEs and can constrain their parameters quite good. In spite of this, they show a larger deviation from the measured signatures than the more complex models Lumped 3 and Semi-Lumped 3. This is most apparent for the signatures regarding the frequency and duration (marked yellow in Fig. 7). In this case, the model Lumped 1 completely fails to get the low flow event duration right (Q_LD_). The model also reveals a large error in the prediction of high flow event duration (Q_HD_) and the low flow exceedance percentiles (Q_99_). To a lesser degree the slope of the flow duration curve (S_FDC_), the low flow variability (Q_LV_), and the high and low flow frequency (Q_LF_, Q_HF_), are also challenging for the model Lumped 1. Contrary, Lumped 2 does have a smaller error in its simulation of its hydrological signatures. This model only has problems in predicting the low and high flow durations (Q_LD_, Q_HD_) and the characteristic recession time at median flow (T_0_). Similarly, Lumped 3 has the same problems as Lumped 2, but is able to get the low flow duration (QLD) more correct. Although, this comes at the cost that it has a larger error in the characteristic recession time at the median flow (T_0_) and the low flow duration (Q_LF_). The Semi-Lumped 3 models with both spatial set ups of vegetation and vegetation/height have overall smaller errors than the lumped models. Nevertheless, they also have problems in getting the low and high flow durations right (Q_LD_, Q_HD_), but to a lesser extent than the lumped models. At the same time, they have smaller errors in the characteristic recession time at the median flow (T_0_), while Lumped 3 fails at that.

All models behave very similar for both PET methods in regard to the hydrological signatures. Only the low flow duration error (Q_LD_) in Lumped 2 is considerably higher for the Penman version, while the low flow frequency error is lower (Q_LF_).

## Discussion

When we look at the model performances as indicated by the KGE (Fig. 3) the two most simple model structures Lumped 1 and Lumped 2 seem to perform fairly well, showing only a very small range of the KGE at a high level, both during the calibration and validation. All other models have much larger spread for their KGE, even though the ROPE algorithm is intended to avoid that^20^. When we compare the KGE values for calibration and validation all models except Lumped 1 perform better in the validation period. A better performance during validation is usually considered as a sign for models of an appropriate complexity, which have an adequate number of parameters^11,38^. However, this might also be caused by less extreme rainfall events or reduced discharge variability in the validation period in comparison to the calibration period^14^. This drop in performance from calibration to validation of Lumped 1 hints that the model is not able to predict well, which often is the case when a model is too simple^39^.

The models, which have a small range for the KGE, also have tightly constrained parameters (Fig. 5). Again, the parameters of the two most simple models (Lumped 1 and Lumped 2) can be constrained most. Lumped 1 has a median parameter constrain of 95%. This is quite high, since other studies with a comparable number of parameters could not constrain their parameters this much^40,41^. However, studies with fewer parameters found similar constraints^42^. This shows that hydrological models with fewer parameters can usually be constrained more easily. Nevertheless, this relationship is not linear and difficult to be generalized. For example, Shen *et al*.^43^ used the SWAT model with twenty parameters and could constrain around half of them while Seibert^44^ was only able to constrain one out of 12 parameters in HBV.

When all models are pooled by the PET method, we could only find large differences in the distributions *ETV1* (volume under which the evapotranspiration is lowered). Therefore, we conclude that the PET method only affects those parameters that are directly related to it. In addition, when the parameter constraint is quantified (Fig. 7) Hargreaves is slightly better for all models. However, the effect is small compared to the strong effects on the parameter values by the PET calculation as also found by other studies^45^.

The main shift in the distribution of the parameters is caused by the switch from the lumped to the semi-lumped model structure (Figs 4, 6). Here, several parameters experience a shift or reshape of their distribution. This is especially the case for *V0_l1* (field capacity of the soil) and *ETV1*. Further, the parameters of the semi-lumped models are less constrained than the parameters in the lumped models (Fig. 5). Nevertheless, they are similar constrained in comparison with models of similar complexity^41,46^. We conclude that the lumped models, especially the more simple ones, are markedly better in constraining the parameters than the more complex models and this can be mainly attributed to the switch from a lumped to a semi-lumped structure.

The patterns found in the hydrological signatures are different to the ones concerning parameter constrainability. Here, the lumped models struggle more than the semi-lumped ones to correctly simulate the hydrological signatures. Especially their ability to simulate the low flows shows larger errors. This is in line with other studies^9,47^ who found that models that do not get the groundwater behavior right or miss a groundwater component fail to simulate discharge minima. Generally, it is stated that models must incorporate as much of the catchments landscape characteristics as possible to come up with reasonable explanatory power^48^ and many studies find a performance increase when switching from a lumped to a semi-distributed model layout^11,49^. Usually, this is attributed to the accounting of rainfall variability^13^ and topography^12^. This might also be the case for the semi-lumped models, as the spatial subdivision might contain a more accurate representation of rainfall. However, there seems to be an upper limit on how much spatial subdivisions make sense for a given amount of data^6,50^, which also seems to be the case for this study. Not much improvement can be found when going from four to eight spatial subdivisions.

Concerning the PET method there seems to be almost no influence on the hydrological signatures (Fig. 7). This is in contrast to other studies^15,51^, who state that getting the PET right is essential to model the discharge successful. The PET method is often attributed to cause large differences between hydrological models^14^. In spite of that, the calculation of the PET might mainly influence the overall water balance, while not having a large effect on the daily discharge. In our study, the Hargreaves and Penman methods were similar enough not to cause any differences between the simulation of the hydrological signatures. The only exception from this is Lumped 2, where the Penman version depicts a larger error in the low flow duration (QLD) and a smaller error in the low flow frequency (QLF). This is caused by the shift in the parameter ETV1 and LAI, which both control the evapotranspiration. The simpler model Lumped 1 has such a large error in its signatures that it overlays the differences between the different PET methods. On the other hand, the more complex models are able to correctly simulate the low flow characteristics due to their more realistic structure.

Overall, the models used in this study show two patterns along their axis of complexity. While the simple models (Lumped 1 and Lumped 2) are quite good at constraining their parameter and not so good at getting hydrological signatures right, it is the other way around for the more complex models (Semi-Lumped 3, both spatial versions). They have problems with constraining their parameters, but manage to have a lower error at their hydrological signatures. This seems counterintuitive, as tightly constrained parameters are seen as a property of good models, but it highlights that is important to use several criteria to evaluated models to avoid one sided results^52^. A better model performance in the calibration than in the validation period is often seen as a sign of an overfitting of the more complex models^2,9,38,53^. This does not apply here as all models perform better in validation. One possible explanation for the good performance of the more complex models concerning the hydrological signatures, can be found in the study of Shen *et al*.^43^. They used a semi-distributed model (SWAT) with twenty parameters and found that they could not constrain most of their parameters. However, they stated that unconstrained parameters do not imply that those parameters are not important for the model, but simply that they interact with other parameters in the model. Similar results were also stated by Zhao *et al*.^54,55^. They also used the SWAT model and found that in such a more complex model set up, the parameters seem more disperse. Still, the added complexity of the model allows SWAT to more accurately reflect the real conditions, but this complexity must be constrained with additional data^55^, like it was done in this study by using information about the land use and topography of the catchment.

This interaction of parameters could be caused by an increase in uncertainty due to the introduction of additional data to the semi-lumped models. Therefore, simple models will not show the reality but merely hide the uncertainties inherent in the data^56^. Hence, models should include additional data like landscape related process heterogeneity^49^, land cover^57^ if possible, as it allows for a more realistic prediction without hiding uncertainties.

Overall, the results in this study show that it is easier to constrain parameters of simple models. However, their simple structure does not allow them to provide realistic simulations. We analysed this behaviour with the ability to simulate hydrological characteristics. It turned out that the simply structured models have strong weaknesses here. For the more complex models, the story is different. Their parameters are harder to constrain, but they outperform the simple models regarding the hydrological characteristics. This indicates a clear trade-off between the ability to constrain the parameters of these models and the ability to realistically simulate the discharge.

## Conclusion

This study explored five hydrological models of differing complexity implemented with two PET methods concerning the trade-offs between parameter constrainability and their ability to simulate hydrological signatures. We used the same model building framework, numerical solver, calibration algorithm, and forcing data to ensure that the results are only influenced by the model structure itself. The results show that parameters of the more complex models are less constrained, still the models have a smaller error in simulating hydrological signatures in comparison with the simpler models. The selection of the PET method only affected canopy parameters, but had hardly any influence on parameters of the flow generating processes. We note that the results depend on the investigated site and period and may not be generalizable. However, the catchment used has typical properties for a Central German Upland catchment and thus the findings should at least be applicable in this region. This study also shows the benefits of comparing model in a modelling framework, as it ensures that all models are handled equal. Finally, this study highlights the importance of not focusing too narrowly on the parameter uncertainty, as models that incorporate more relevant hydrological processes are able to simulate a river more realistically concerning hydrological signatures, even though their parameters are less constrained.

## Supplementary information

Supplementary Information for: Trade-offs between parameter constraints and model realism: a case study

## Data Availability

Datasets are available by contacting the Hessian Agency for Nature Conservation, Environment and Geology (HLNUG) (https://www.hlnug.de/service/english.html, last access: 23 August 2018).

## Code Availability

The code of all hydrological models can be found at GitHub^28^.
