# Author Correction: Trade-offs between parameter constraints and model realism: a case study

**DOI:** 10.1038/s41598-022-09089-w

**Published:** 2022-03-23

**Authors:** Florian U. Jehn, Alejandro Chamorro, Tobias Houska, Lutz Breuer

**Affiliations:** 1 Institute for Landscape Ecology and Resources Management (ILR), Research Centre for BioSystems, Land Use and Nutrition (iFZ), Justus Liebig University Giessen, Heinrich-Buff-Ring 26, 35390 Giessen, Germany; 2 Centre for International Development and Environmental Research (ZEU), Justus Liebig University Giessen, Senckenbergstrasse 3, 35392 Giessen, Germany

Correction to: *Scientific Reports* 10.1038/s41598-019-46963-6, published online 24 July 2019

This article contains an incorrect email address for Florian U. Jehn. Correspondence and requests for materials should be addressed to florianjehn@posteo.de.

