# Supplementary material for: Trade-offs between parameter constraints and model realism: a case study
Source: Sci Rep. 2019 Jul 24;9:10729. doi: 10.1038/s41598-019-46963-6 (PMC6656744; doi:10.1038/s41598-019-46963-6)
Supplement: Supplementary file 1 — Supplementary Information for: Trade-offs between parameter constraints and model realism: a case study [file 41598_2019_46963_MOESM1_ESM.docx]

Supplementary Information for:

Trade-offs between parameter constraints and model realism: a case study

Florian U. Jehn^1 *^, Alejandro Chamorro^1^, Tobias Houska^1^, Lutz Breuer^1,2^

^1^Institute for Landscape Ecology and Resources Management (ILR), Research Centre for BioSystems, Land Use and Nutrition (iFZ), Justus Liebig University Giessen, Heinrich-Buff-Ring 26, 35390 Giessen, Germany

^2^Centre for International Development and Environmental Research (ZEU), Justus Liebig University Giessen, Senckenbergstrasse 3, 35392 Giessen, Germany

Supplementary Information includes:

Description of the applied methods for the potential evapotranspiration

## Detailed description of the applied methods for the potential evapotranspiration.

The Hargreaves methods uses mean, min and max temperature as input data (equation 2) and is thus a relatively simple temperature based approach.

$ET=0.0135{\cdot K}_{t}{\cdot s}_{0}\cdot\sqrt{\Delta T}\cdot(T+17.8)$ (2)

Where

ET = reference evapotranspiration [mm day^-1^]

$K_{t}$ = $0.00185\cdot\Delta T^{2}-0.0433\cdot\Delta T+0.4023$ continentality factor

$\Delta$ = daily temperature range [K] $T_{max}- T_{min}$

T = daily mean temperature [°C]

$s_{0}$ = extra-terrestrial solar radiation [mm day^-1^]$15.392\cdot d_{r}\cdot(\omega_{s}\cdot\sin\left( \Phi\right)\cdot\sin\left( \gamma\right)+\cos\left( \Phi\right)\cdot\cos\left( \gamma\right)\cdot\sin\left( \omega_{s} \right))$

$d_{r}$ = $1+0.0033\cdot\cos(DOY\frac{2\pi}{365})$ relative distance between earth and sun

$\omega_{s}$ = $\cos^{-1} (-\tan(\Phi)\cdot\tan(\gamma))$ sunset hour angle radians

$\gamma$ = $0.4039\cdot\sin(DOY\frac{2\pi}{365}-1.405)$ solar declination radians

Φ = geographic latitude [radians]

DOY = day of year

The Penman-Monteith model uses further meteorological data (equation 3) apart from temperature. This is classified as a combined approach for estimating PET ^17^ and is usually regarded as a more realistic bio-physically based method ^16^. However, it has been stated that this only holds true if forcing data is of good quality ^65^. The implementation used here is the widely known FAO version ^30^.

$\lambda ET= \frac{\Delta\left( R_{n}-G \right)+ p_{a}\cdot c_{p}\cdot\frac{e_{s}- e_{a}}{r_{a}}}{\Delta+ \gamma\cdot(1+ \frac{r_{s}}{r_{a}})}$ (3)

Where

$\Delta$ = $4098 \cdot\frac{0.6108 \cdot e^{12.27T}}{\left( T+237.3 \right)^{2}} \cdot\frac{kPa}{^{\circ}C}$

T = Actual Temperature [°C]

$R_{n}$ = net Radiation [$\frac{MJ}{m^{2}\cdot day}]$

G = $0 \left[ \frac{MJ}{m^{2} \cdot day} \right]$ if daily average

$0.1 R_{n}$ if day time

$0.5 R_{n}$if night time

$\gamma$ =$\frac{c_{p}P}{\epsilon\lambda}$: Psychrometric constant $\left[ \frac{kPa}{^{\circ}C} \right]$

$c_{p}$ = 0.001013 $\left[ \frac{MJ}{kg \cdot^{\circ}C} \right]$ specific heat at constant pressure

$P$ = 101.3$\cdot(\frac{293-0.0065z}{293}$)^5.26^

$\epsilon$ = $0.622 \left[ \frac{mol / g vapor}{mol / g liquid water} \right]$

$\lambda$ = $2.45 \left[ \frac{MJ}{kg} \right]$

$R$ = $0.287 \left[ \frac{kJ}{kg k} \right]$ Specific gas constant

$p_{a}$ = $\frac{P}{1.01 \cdot\left( T+273 \right)\cdot R}$

$e_{s}$ = Saturated vapour pressure $[kPa]$

$e_{a}$ = Actual vapour pressure $[kPa$]

$r_{a}$ = $\frac{\ln\left( \frac{2-d}{0.123h} \right) \cdot\ln(\frac{2-d}{0.0123h})}{{0.41}^{2} \cdot u_{2}}$

$h$ = Vegetation height [$m]$

$u_{2}$ = Wind speed in 2 m above canopy [$\frac{m}{s}]$

$r_{s}$ = $\frac{100 \frac{m}{s}}{0.5 LAI} \frac{s}{m}$
